# One step closer to understanding the role of bacteria in diabetic foot ulcers: characterising the microbiome of ulcers

**DOI:** 10.1186/s12866-016-0665-z

**Published:** 2016-03-22

**Authors:** Karen Smith, Andrew Collier, Eleanor M. Townsend, Lindsay E. O’Donnell, Abhijit M. Bal, John Butcher, William G. Mackay, Gordon Ramage, Craig Williams

**Affiliations:** School of Health, Nursing and Midwifery, Institute of Healthcare Associated Infection, University of the West of Scotland, Barbour Building, Paisley Campus, Paisley, PA1 2BE UK; NHS Ayrshire and Arran, University Hospital Ayr, Ayr, UK; Infection and Immunity Research Group, Glasgow Dental School, School of Medicine, College of Medical, Veterinary and Life Sciences, University of Glasgow, Glasgow, UK

**Keywords:** Diabetes, Diabetic foot ulcer, Infection, Microbiome, Next generation sequencing, 16S amplicon sequencing

## Abstract

**Background:**

The aim of this study was to characterise the microbiome of new and recurrent diabetic foot ulcers using 16S amplicon sequencing (16S AS), allowing the identification of a wider range of bacterial species that may be important in the development of chronicity in these debilitating wounds. Twenty patients not receiving antibiotics for the past three months were selected, with swabs taken from each individual for culture and 16S AS. DNA was isolated using a combination of bead beating and kit extraction. Samples were sequenced on the Illumina Hiseq 2500 platform.

**Results:**

Conventional laboratory culture showed positive growth from only 55 % of the patients, whereas 16S AS was positive for 75 % of the patients (41 unique genera, representing 82 different operational taxonomic units (OTU’s). *S. aureus* was isolated in 72 % of culture-positive samples, whereas the most commonly detected bacteria in all ulcers were *Peptoniphilus* spp., *Anaerococcus* spp. and *Corynebacterium* spp., with the addition of *Staphylococcus* spp. in new ulcers. The majority of OTU’s residing in both new and recurrent ulcers (over 67 %) were identified as facultative or strict anaerobic Gram-positive organisms. Principal component analysis (PCA) showed no difference in clustering between the two groups (new and recurrent ulcers).

**Conclusions:**

The abundance of anaerobic bacteria has important implications for treatment as it suggests that the microbiome of each ulcer “starts afresh” and that, although diverse, are not distinctly different from one another with respect to new or recurrent ulcers. Therefore, when considering antibiotic therapy the duration of current ulceration may be a more important consideration than a history of healed ulcer.

## Background

A serious complication of diabetes is the development of foot ulcers. Patients with diabetes are believed to have a 12–25 % lifetime risk of developing a foot ulcer [1]. The aetiology of diabetic foot ulceration is complex. Foot ulcers often develop due to a combination of intrinsic factors, such as peripheral neuropathy, poor extremity perfusion, foot deformity, changes to the plantar foot soft tissues plus extrinsic mechanical factors, such as high plantar pressures [2]. Diabetic foot ulcers have a significant negative impact on health and quality of life and are the most common cause of hospitalisation in patients with diabetes [3].

Diabetic foot ulcers often heal very slowly, due to diabetes-associated micro-vascular disease and impaired host immune response, and these open wounds provide a niche for infection [4, 5]. Bacteria can exist within the wound as multi-layered microbial communities, known as biofilms, surrounded by a self-produced protective extracellular ‘slime’ [6]. The presence of a biofilm makes infections very difficult to resolve, as the structure shields the encased cells from antimicrobial agents and the host immune system, allowing bacteria to persist and impair healing [7]. Many foot ulcers fail to heal and cause serious complications, such as osteomyelitis. Infection is the most common cause of lower limb amputation in diabetic foot ulcers [3]. In the UK, more than one hundred amputations are carried out each week in individuals with diabetes [1]. In addition to the significant trauma, the cost to the NHS of treating infected diabetic foot ulcers and resulting amputations is estimated to be in the region of £900 million per year [8].

Standard treatment of diabetic foot ulcers involves debridement of the necrotic tissue, management of infection and off-loading of the ulcer [9]. Infection is routinely confirmed by laboratory culture of bacteria in a swab taken from the wound. Culture-dependent methods show bias towards microorganisms that are able to grow well on laboratory culture media. More fastidious organisms may not be identified, resulting in a delay in the appropriate treatment [10, 11]. Molecular methods are advancing and becoming more accessible and affordable (including 16S amplicon sequencing [16S AS]), and it is now possible to use bacterial DNA from the wound site to identify the pathogens present [12]. A greater understanding of both the bacteria present in diabetic foot ulcers and how these bacteria interact with one another, and the host, will be crucial for the development of reliable models of infection and effective treatments.

It was our hypothesis that the specific microbiome associated with new and recurrent diabetic foot ulcers differed, and that this impacted the ability to effectively manage these wounds. Therefore, the aim of this study was to use 16S AS technologies to characterise the microbiome of new and recurrent ulcers and to undertake comparative analyses to gain understanding of whether the presence of certain microorganisms were associated with the inability of an ulcer to heal.

## Results

### The detection of bacteria in diabetic foot ulcers by conventional laboratory culture and molecular methods

By conventional laboratory culture, 11 samples (55 %) were positive for microbial growth by standard aerobic and anaerobic culture methods (4 recurrent ulcer samples and 7 new ulcer samples) (Table 1). The remaining 9 swab samples (45 %) produced no significant growth (6 recurrent and 3 new ulcer samples). In the samples that were positive for bacterial growth, 6 contained more than one species. *S. aureus* was isolated in 8 of culture-positive samples, anaerobes were isolated from 4 samples, beta-haemolytic streptococci from 2 samples, and *Candida* spp. was identified alone in only 1 sample. One control swab sample collected from the healthy skin (sample 19) showed a moderate growth of *S. aureus*. These results are summarised in Table 1. Moreover, *S. aureus* was confirmed in 50 % of the samples tested by PCR. Spearman’s rank correlation showed that this correlated to culture results (*p* < 0.05, CI 0.5761–0.9642).

**Table 1:** Clinical laboratory culture of diabetic foot wound samples

Sample number	New or recurrent ulcer	Bacteria isolated
1	Recurrent	NSG^a^
2	Recurrent	NSG
3	Recurrent	*Candida spp.*
4	New	Mixed growth + anaerobe
5	New	Mixed growth + anaerobe
6	Recurrent	*S. aureus* + anaerobe
7	New	*S. aureus* + anaerobe
8	New	NSG
9	New	*S. aureus* + beta-haemolytic *Streptococcus* (Group G)
10	Recurrent	NSG
11	New	NSG
12	New	*S. aureus*
13	New	*S. aureus* + beta-haemolytic *Streptococcus* (Group G)
14	Recurrent	*S. aureus*
15	Recurrent	NSG
16	Recurrent	NSG
17	New	*S. aureus*
18	New	NSG
19	Recurrent	*S. aureus*
20	Recurrent	NSG

^a^NSG - No significant growth

### Microbiome analysis diabetic foot ulcers by 16S amplicon sequencing

DNA was successfully amplified and sequenced from 16 of the samples (75 %), including 9 from new ulcers and 7 from recurrent ulcers. These 16 samples produced an average of 1,767,142 sequence reads, which were filtered for quality and assigned an OTU using a minimum sequence similarity of 97 %. The abundance of each species in wound samples of greater than 0.5 % was reported. Raw data sets are available from the following website: http://www.ncbi.nlm.nih.gov/bioproject/304940.

**Fig. 1:**
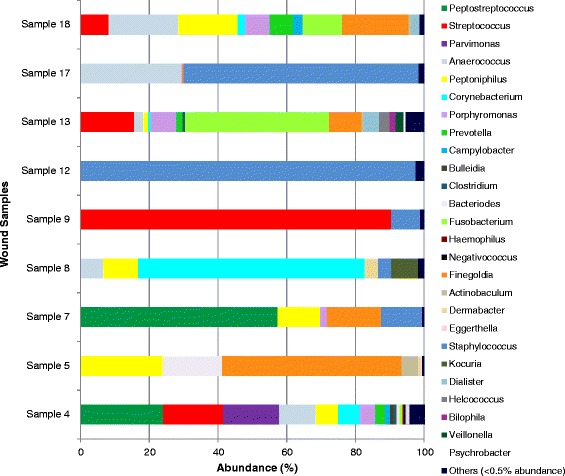
Abundance (%) of bacterial genus within new diabetic foot ulcers

**Fig. 2:**
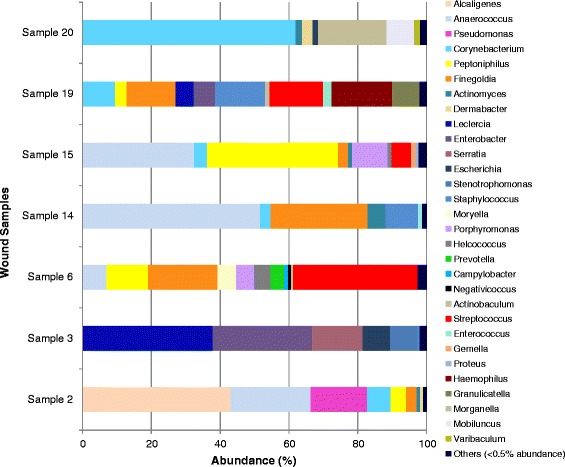
Abundance (%) of bacterial genus within recurrent diabetic foot ulcers

**Fig. 3:**
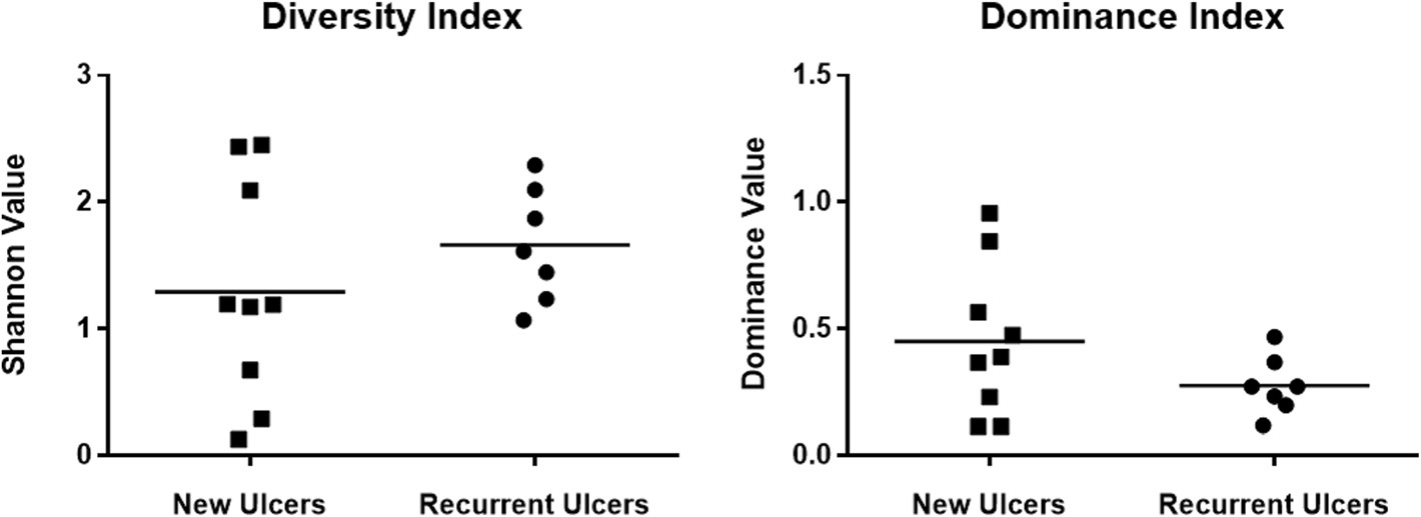
Dominance and diversity (*Shannon Value*) indices for both sets of ulcers

In the 16 ulcer samples there were 41 unique genera, representing 82 different OTU’s, which ranged from 21 different species (sample 4) to only 2 species (sample 9). The species identified in new and non-healing ulcers are displayed in Table 2, along with their frequency of detection. In new ulcers there were 94 different OTU’s identified; most frequently detected genera were *Peptoniphilus* (6 samples), *Staphylococcus* (5 samples), *Anaerococcus* (5 samples) and *Corynebacterium* (4 samples)*.* In recurrent ulcers 73 unique OTU’s were identified; the most frequently detected genera were *Corynebacterium* (5 samples), *Peptoniphilus* (4 samples) and *Anaerococcus* (4 samples). The relative abundance (%) of each genus is displayed in Fig. 1 for new ulcers and Fig. 2 for recurrent ulcers. Dominance and Diversity indices indicated a increase in diversity on the recurrent ulcers (Fig. 3), while the new ulcers had higher levels of dominance. However, these differences were not statistically significant (unpaired *t*-test, Shannon value *p* = 0.3287, Dominance value *p* = 0.1649).

**Table 2:** List of species identified in the new and recurrent ulcers by 16S AS

Species identified	No. of samples		Gram type	Oxygen tolerance
	New	Recurrent		
*Actinobaculum massiliense*	1	0	Gram Positive	Facultative anaerobe
*Actinobaculum schaalii*	2	2	Gram Positive	Facultative anaerobe
*Actinomyces europaeus*	0	1	Gram Positive	Facultative anaerobe
*Actinomyces hominis*	0	1	Gram Positive	Facultative anaerobe
*Actinomyces neuii*	0	1	Gram Positive	Facultative anaerobe
*Actinomyces radingae*	0	1	Gram Positive	Facultative anaerobe
*Alcaligenes faecalis*	0	1	Gram Negative	Aerobe
*Anaerococcus murdoch*	3	2	Gram Positive	Anaerobe
*Anaerococcus tetradius*	1	0	Gram Positive	Anaerobe
*Anaerococcus vaginalis*	5	4	Gram Positive	Anaerobe
*Bacteriodes fragilis*	2	0	Gram Negative	Anaerobe
*Bilophila wadsworthia*	1	0	Gram Negative	Anaerobe
*Bulleidia extructa*	1	0	Gram Positive	Anaerobe
*Campylobacter ureolyticus*	2	1	Gram Negative	Anaerobe
*Clostridium saccharogumia*	2	0	Gram Positive	Anaerobe
*Corynebacterium accolens*	0	1	Gram Positive	Facultative anaerobe
*Corynebacterium amycolatum*	4	0	Gram Positive	Facultative anaerobe
*Corynebacterium aurimucosum*	2	1	Gram Positive	Facultative anaerobe
*Corynebacterium freiburgense*	0	1	Gram Positive	Facultative anaerobe
*Corynebacterium hansenii*	1	0	Gram Positive	Facultative anaerobe
*Corynebacterium mycetoide*	0	1	Gram Positive	Facultative anaerobe
*Corynebacterium simulans*	1	3	Gram Positive	Facultative anaerobe
*Corynebacterium tuberculostearicum*	1	0	Gram Positive	Facultative anaerobe
*Corynebacterium xerosis*	1	0	Gram Positive	Facultative anaerobe
*Dermabacter hominis*	2	2	Gram Positive	Facultative anaerobe
*Dialister propionicifaciens*	1	0	Gram Negative	Anaerobe
*Dialister micraerophilus*	1	0	Gram Negative	Anaerobe
*Dialister pneumosintes*	1	0	Gram Negative	Anaerobe
*Eggerthella lenta*	1	0	Gram Positive	Anaerobe
*Enterobacter hormaechei*	0	2	Gram Negative	Facultative anaerobe
*Enterococcus canintestini*	0	2	Gram Negative	Facultative anaerobe
*Escherichia fergusonii*	0	1	Gram Negative	Facultative anaerobe
*Escherichia vulneris*	0	1	Gram Negative	Facultative anaerobe
*Finegoldia magna*	5	5	Gram Positive	Anaerobe
*Fusobacterium canifelinum*	1	0	Gram Negative	Anaerobe
*Fusobacterium nucleatum*	1	0	Gram Negative	Anaerobe
*Fusobacterium periodontium*	1	0	Gram Negative	Anaerobe
*Gemella morbillorum*	0	1	Gram Positive	Anaerobe
*Granulicatella adiacens*	0	1	Gram Positive	Facultative anaerobe
*Haemophilus parainfluenzae*	1	1	Gram Negative	Facultative anaerobe
*Helcococcus kunzii*	1	2	Gram Positive	Anaerobe
*Kocuria atrinae*	1		Gram Positive	Anaerobe
*Leclercia adecarboxylata*	0	2	Gram Negative	Facultative anaerobe
*Mobiluncus curtisii*	0	1	Gram Positive	Anaerobe
*Morganella morganii*	0	1	Gram Negative	Facultative anaerobe
*Moryella indoligenes*	0	1	Gram Positive	Anaerobe
*Negativicoccus succinicivorans*	1	1	Gram Negative	Anaerobe
*Parvimonas micra*	1	0	Gram Positive	Anaerobe
*Peptoniphilus gorbachii*	4	4	Gram Positive	Anaerobe
*Peptoniphilus ivorii*	2	3	Gram Positive	Anaerobe
*Peptoniphilus lacrimalis*	2	1	Gram Positive	Anaerobe
*Peptoniphilus olsenii*	2	0	Gram Positive	Anaerobe
*Peptostreptococcus anaerobius*	2	0	Gram Positive	Anaerobe
*Peptostreptococcus stomatis*	1	0	Gram Positive	Anaerobe
*Porphyromonas asaccharolytica*	3	2	Gram Negative	Anaerobe
*Porphyromonas bennonis*	2	1	Gram Negative	Anaerobe
*Porphyromonas somerae*	2	2	Gram Negative	Anaerobe
*Porphyromonas uenonis*	1	1	Gram Negative	Anaerobe
*Prevotella bergensis*	1	1	Gram Negative	Anaerobe
*Prevotella buccalis*	2	0	Gram Negative	Anaerobe
*Prevotella corporis*	1	0	Gram Negative	Anaerobe
*Prevotella intermedia*	1	0	Gram Negative	Anaerobe
*Prevotella timonensis*	1	1	Gram Negative	Anaerobe
*Proteus myxofaciens*	0	1	Gram Negative	Anaerobe
*Pseudomonas indica*	0	1	Gram Negative	Aerobe
*Pseudomonas otitidis*	0	1	Gram Negative	Aerobe
*Psychrobacter lutiphocae*	1	0	Gram Negative	Aerobe
*Serratia grimesii*	0	1	Gram Negative	Facultative anaerobe
*Staphylococcus carnosus*	1	0	Gram Positive	Facultative anaerobe
*Staphylococcus chromogenes*	2	0	Gram Positive	Facultative anaerobe
*Staphylococcus devriesei*	1	0	Gram Positive	Facultative anaerobe
*Staphylococcus hominis*	5	2	Gram Positive	Facultative anaerobe
*Staphylococcus pettenkoferi*	1	0	Gram Positive	Facultative anaerobe
*Staphylococcus saprophyticus*	0	1	Gram Positive	Facultative anaerobe
*Stenotrophomonas pavanii*	0	1	Gram Negative	Aerobe
*Streptococcus agalactiae*	2	0	Gram Positive	Facultative anaerobe
*Streptococcus anginosus*	0	1	Gram Positive	Facultative anaerobe
*Streptococcus canis*	1	0	Gram Positive	Facultative anaerobe
*Streptococcus dysgalactiae*	1	0	Gram Positive	Facultative anaerobe
*Streptococcus infantarius*	1	2	Gram Positive	Facultative anaerobe
*Varibaculum cambriense*	0	1	Gram Positive	Anaerobe
*Veillonella dispar*	1	0	Gram Negative	Anaerobe
*Veillonella rogosae*	1	0	Gram Negative	Anaerobe

**Fig. 4:**
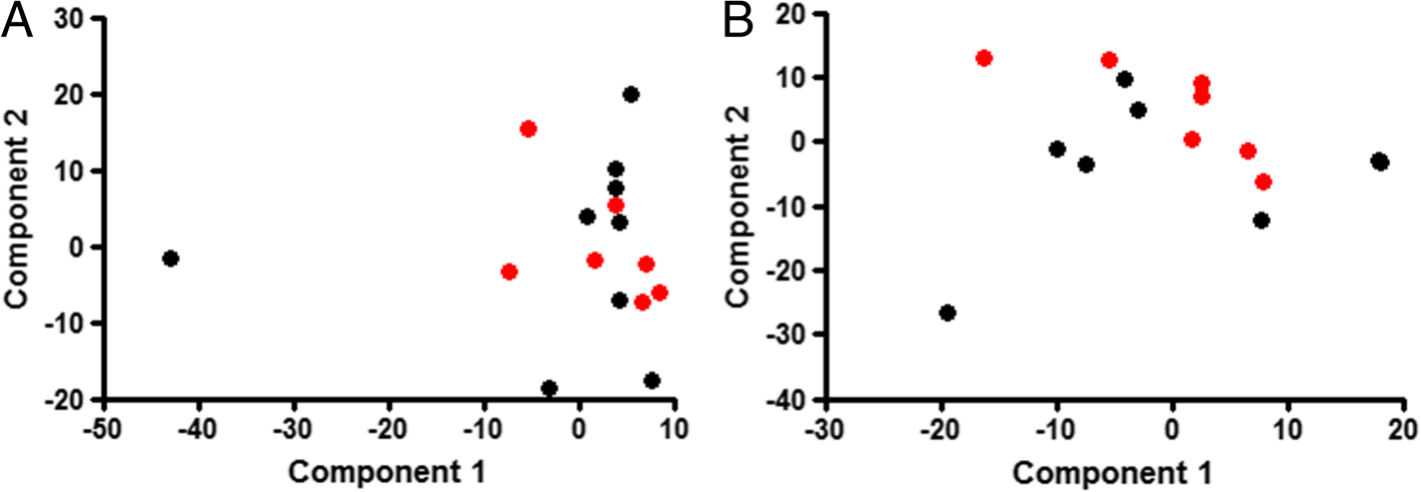
Principal Component Plots. New ulcers (*black circles*) and recurrent ulcers (*red circles*) were plotted with **a** and (**b**) without sample 13

The majority of OTU’s residing in both new and recurrent ulcers (over 67 %) were identified as Gram-positive organisms. These were mostly Gram-positive cocci, such as *Staphylococcus*, *Streptococcus*, *Anaerococcus*, *Peptoniphilus* and *Finegoldia.* However, the Gram-positive rods, *Corynebacterium*, *Clostridium* and *Actinomyces* were also frequently detected. In both types of ulcers the most frequently identified Gram-negative organisms were *Porphyromonas* spp. In all sixteen ulcers sampled the majority of species identified by 16S AS were classed as facultative or strict anaerobes. In newly formed ulcers, only one of the 94 OTU’s identified (1.06 %) was an aerobe and in recurring ulcers, 4 of the 73 OTU’s identified (5.48 %) were aerobes. The results at OTU’s level are outlined in Table 2. Comparison of the bacterial classes and genera within each group showed a significant difference (*p* < 0.05) between the amount of Gammaproteobacteria in the two groups. There were no other significant differences. In principal component (PC) analysis (Fig. 4), PC1 was significantly different (*p* = 0.0229) between the two groups once the outlier sample (sample 13) was removed. However, we were unable to determine which bacteria are contributing to this difference due to a lack of clear clusters in graphical format.

### Ulcer and clinical characteristics correlations

Both HbA1c and the duration of the patient’s diabetes had correlated with dominance (*p* = 0.0174) and diversity (*p* = 0.0168) values. A lower HbA1c value and shorter duration of diabetes correlated with the higher diversity (lower dominance statistic and higher Shannon value) within the ulcer. No other ulcer characteristics, including predominant genera identified by 16S AS, number of OTUs, oxygen tolerance and bacterial morphology, had any correlation with the patient’s characteristics.

## Discussion

Diabetic foot ulcers are a common complication of poorly controlled diabetes and are a significant cause of morbidity and hospitalisation in sufferers of this disease [3]. These debilitating wounds heal slowly and in severe cases, lower extremity amputation may be the only clinical option [1]. For many years the role of bacteria in chronic wound healing was often overlooked, as approximately half of diabetic foot ulcers exhibit no clinical evidence of infection [13]. However, many individuals with diabetes have an impaired inflammatory response and may not show the classical signs of infection in a wound with a high microbial burden [14]. The concept that non-healing in chronic wounds is associated with bacterial load was introduced [5, 15], and bacterial colonisation and proliferation within diabetic foot ulcers is now believed to significantly retard wound healing. Therefore, a greater understanding of the microbiome of these chronic wounds is urgently required to help guide the successful treatment [16]. Here we report for the first time the complex microbiome of new and recurrent ulcers, demonstrating how diverse polymicrobial biofilm populations are common in diabetic patients. This is of significance as there is growing evidence that polymicrobial interactions may synergise the pathogenic potential of one or other microorganism [17]. This has implications for patient management, as eradication of microorganisms could be important in controlling these chronic wounds [7, 18, 19].

In the clinic, infection is generally suspected based on the presence of at least two classic signs of inflammation (erythema, warmth, tenderness, pain or induration) or purulent secretions [20]. The standard method of diagnosis is by traditional laboratory culture of a sample taken directly from the wound. Our study relied on swab sampling rather than wound biopsy because there was a desire to limit the use of invasive procedures. The presence of bacteria was detected in ten of the twenty swab samples cultured. All other samples produced no significant growth. Standard laboratory reporting only provided basic information and did not readily identify the precise species present in the majority of samples, though *S. aureus* was the most commonly isolated pathogen (40 % of samples). It has been well documented in studies of diabetic foot ulcers that *S. aureus* is the most commonly detected pathogen by laboratory culture [21, 22]. The frequent identification may be due to the ability of staphylococci to grow under normal laboratory conditions when these methods often fail to identify slow-growing, fastidious or anaerobic organisms [23]. This does not mean that these organisms are an insignificant coloniser of chronic wounds. In a retrospective study, it was found that 79 % of wounds sampled were infected with *S. aureus* [21]. More alarmingly, 30 % of these isolates were methicillin resistant *S. aureus* (MRSA). In this study 7 of the 8 *Staphylococcus* isolates detected in our study were resistant to penicillin in culture, and 3 of these isolates were positive for *mecA* by PCR. Therefore, culture-based methods still play an important role in patient management, but do not necessarily give a true representation of the pathogenic burden.

Conventional culture techniques have a tendency to produced false negative results, with over 37 % of samples showing no signs of infection by culture alone. It is now widely accepted that past reliance on standard culture techniques has led to an underestimation of the microbiome of chronic wounds, only detecting approximately 1 % of the inhabiting bacteria, which is biased by selective culture [16, 23]. Recent studies using molecular methods have confirmed that chronic wounds, including diabetic foot ulcers, have a polymicrobial nature instead of being colonised by a single species [24]. In this study, the number of OTU’s in a new ulcer samples ranged from 2 to 21, and in recurrent ulcers species ranged from 6 to 17. There is growing evidence that, as with other persistent infections, the bacteria that reside within chronic wounds grow within biofilm communities [18, 19, 25]. This was supported by studies utilising microscopy that have shown that specimens from 60 % of chronic wounds contained polymicrobial biofilm structures [6]. The presence of bacterial cells encased within a biofilm may contribute to the chronicity of infection, as biofilm-associated cells are notoriously recalcitrant. Poor penetration of the biofilm structure and extracellular matrix, nutrient limitation leading to slow growth and phenotypic variants, protect the cells from the effects of antimicrobials and the host immune response [7, 18]. The eradication of polymicrobial biofilms within diabetic foot ulcers could key to resolving these chronic wounds.

The analysis of the microbiome of this patient group showed that the most frequently identified genera were the Gram-positive facultative anaerobes, *Staphylococcus* and *Corynebacterium*. These organisms are part of the normal microbiota of healthy skin particularly in moist areas, such as the foot [26, 27]. Dowd et al. hypothesised the concept that individual bacterial species may not be able to maintain a pathogenic biofilm alone, but in a symbiotic polymicrobial community in a DFU, pathogenic biofilm may form [24]. Therefore, although these bacteria are normally commensals, they may be contributing to a pathogenic community. In our study, *S. aureus* was not detected by 16S AS, instead all species identified were coagulase-negative staphylococci (CoNS). We confirmed the presence of *S. aureus* DNA in these samples using PCR, so while 16S AS is undoubtedly robust at the genus level results expressed at species level should be interpreted with some caution. This underlines the current problem with using data from 16S AS at the species level [28]. In the past, CoNS and *Corynebacterium* spp. have been dismissed as contaminants of normal skin flora in diabetic foot ulcers, but several studies have highlighted their importance as potential pathogens. In the case of CoNS, there is a link between the presence of these species in diabetic foot ulcers and the incidence of osteomyelitis [29, 30]. Armstrong et al. (1995) studied a predominantly diabetic patient group with osteomyelitis and found that 40 % of bone cultures were positive for CoNS, 63 % of which were resistant to the antibiotic methicillin [29]. A high incidence of *Corynebacterium* spp. has also been reported in studies using both culture and molecular tools to analyse the bio-burden of diabetic foot ulcers [24, 31, 32], and combined with our findings highlights the importance of CoNS and *Corynebacterium* spp. in relation to chronic wounds, particularly in individuals with diabetes who may have an impaired immune response [22, 29–32].

In the biological niche of the diabetic ulcer the combination of necrotic tissue and low oxygen tension promotes the proliferation of facultative or obligate anaerobes [14, 33]. The majority of OTU’s identified in our study conform to this logic, with obligate anaerobes making up 65.9 % of the OTU’s in new ulcers and 56.1 % in recurrent ulcers. Only 1 OTU detected in new ulcers and 4 OTU’s in recurrent ulcers were aerobic organisms. By culture our study was only able to identify anaerobes in 25 % samples tested but by 16S AS anaerobes were found in 87.5 % of samples screened. The most frequently identified anaerobes in new ulcers were in the genera *Peptoniphilus* (6/9 new ulcers), *Anaerococcus* (5/9 new ulcers), *Finegoldia* (5/9 new ulcers), *Porphyromonas* (4/9 new ulcers), and *Prevotella* (3/9 new ulcers). In recurring ulcers the most frequently identified anaerobes were also *Finegoldia* (5/7 recurring ulcers), *Peptoniphilus* (4/7 recurring ulcers), *Anaerococcus (*4/7 recurring ulcers), *Porphyromonas* (2/7 recurring ulcers), with the addition of *Actinomyces* (4/7 recurring ulcers), which was not detected in new ulcers. The obligate anaerobes occurred as part of a polymicrobial microbiome in all cases, and in previous studies this has earned them the name of ‘co-pathogens’, playing down their importance [20]. The fact that anaerobes were detected in over 87 % of the ulcers screened in our study suggests that they may have a much more important role. Our data agrees with three previous molecular studies of chronic wounds which discovered a high incidence of anaerobic organisms including *Anaerococcus, Finegoldia* and *Peptoniphilus* [11, 24, 32, 34]. Although wounds are generally exposed to air, anaerobes may be able to survive if they co-aggregate with facultative anaerobes or aerobe within a polymicrobial biofilm structure, which would protect them from the harmful effects of oxygen and allow them to thrive [35]. The virulence of anaerobic organisms has also recently been highlighted in a study of *Finegoldia magna*, found in 62.5 % of ulcers in our study, which revealed that this pathogen produces an extracellular serine protease (SufA) to degrade collagen in the skin basement membrane [36]. Using such mechanisms, anaerobes may be responsible for much of the pathogenesis associated with chronic diabetic foot ulcers.

The levels of Gammaproteobacteria in the two groups were also found to be significantly different, with the recurrent group having a higher level. This class of bacteria includes *Enterobacteriaceae* and *Pseudomonadales*. Our findings correlate with a culture-based study that reported a high incidence of members of the *Pseudomonas*, *Enterococcus* and *Enterobacteriaceae* groups in moderate to severe diabetic foot ulcers [22]. It is well documented that *Pseudomonas aeruginosa* is frequently isolated from infected diabetic foot ulcers where it is thought to play a role in severe tissue damage [37]. Clinical isolates of this organism collected from chronic wounds may also be multi-drug resistant which makes them very difficult to eradicate with antibiotic therapy [37]. In a longitudinal study of wound microbiota in an animal diabetic ulcer model it was reported that as time progressed there was a significant shift in bacterial type from Furmicutes to genera including Enterobacter, which produced a corresponding decline in wound healing [26, 38]. This shift towards the presence of enteric-types of bacteria in the recurrent wound may be a result of self-colonisation from another body site. No other significant differences were found between the two groups, including in diversity (Fig. 3). There was no difference between the two groups in bacteria with different oxygen tolerance or number of OTU. There was a significant difference in the number of Gram-positives in each group, the recurrent group having less Gram-positive bacteria. When further broken down into morphological types this difference was no longer significant. This difference in Gram positive/negative balance could be accounted for by the difference in levels of Gammaproteobacteria. Additionally PCA analysis (Fig. 4) indicated no formation of distinct groups. Further longitudinal investigations monitoring the ulcer microbiome over time will help determine if the presence of certain species are associated with the chronicity of these wounds and the inhibition of healing.

As lower HbA1c levels and a shorter duration of diabetes correlated with higher diversity within the ulcer this suggests that poorly glycaemic control and persistent diabetes causes a dominant bacterial species to rise within the ulcer. In contrast, Gardner et al. found that there was no link between high HbA1c levels and diversity, but instead poor glycaemic control was linked to a higher abundance of Staphylococci and Streptococci [11].

Optimal treatment of infection relies on accurate diagnosis of the microbes present and delivery of appropriate antimicrobial treatment. Failure to effectively treat the infection in diabetic foot ulcers leads to progressive tissue damage, disrupted wound healing, and serious complications such as osteomyelitis [39]. Due to the reliance on traditional laboratory culture, many clinics underestimate wound flora, as highlighted by our study and others, who compared laboratory culture with molecular methods, discovering that in 45 % of cases an inappropriate antibiotic was prescribed [33]. Clinical Practice Guidelines (2012) recommend that only diabetic foot ulcers with clinical signs of infection are treated with antibiotics due to the adverse effects, financial costs and increasing risk of antibiotic resistance [20]. There have been reports that individuals with diabetic foot ulcers treated with antibiotics, in the absence of clinical signs of infection, exhibited significantly improved rates of healing in comparison to those who did not undergo antibiotic therapy [40]. The clinical guidelines do recommend that patients with signs of mild to moderate infection receive antibiotic therapy targeting aerobic Gram-positive cocci [20]. In our study, the majority of bacteria detected in both new and recurring ulcers were Gram-positive facultative or obligate anaerobes, which would require antibiotics with a wider spectrum of activity to successfully resolve the infection. Metronidazole is the drug of choice to effectively treat a range of infections caused by anaerobic bacteria and it may have an important role to play in the management of chronic diabetic foot ulcer infection [41], though the evidence of its effectiveness in these infections is questionable [42, 43]. The clinical guidelines also stress that definitive therapy be based on obtaining appropriate culture results from the clinical laboratory, however we have shown that the use of clinical culture alone may severely underestimate the microbial load within ulcers and cause a delay in appropriate treatment. Culture independent molecular methods, such as PCR, have revolutionized many areas of clinical microbiology [34] and it is crucial that these are applied to the routine monitoring of infection in diabetic foot ulcers to diagnose the pathogens responsible and guide appropriate treatment, focussing on disruption of the biofilm.

## Conclusions

Due to the minimal differences found in this study between the bacterial colonisation of new and recurrent, there is no need to treat with more severe methods for a secondary ulcer, even though it is associated with a higher risk of infection [20]. Due to minimal differences between the bacterial colonisation of new and recurrent ulcers, there is no need to treat with more severe methods for a secondary ulcer, even though it is associated with a higher risk of infection [20].

Our study produced three key findings:

1. The complexity of the bacterial population present in diabetic foot ulcers is much greater than would be expected from culture studies alone.
2. There is no significant difference in the bacterial populations in new and recurrent ulcers, suggesting that each wound provides a blank canvas for the development of a unique microbiome within each diabetic foot ulcer.
3. 16S AS cannot currently identify all organisms reliably at a species level and this must be taken into consideration when using this technique to characterise the microbiome of an infection site.

Greater understanding of the diabetic foot ulcer microbiome will help guide new strategies to effectively control the growth of polymicrobial biofilms and improve healing, directly benefiting patients suffering from these debilitating wounds.

## Methods

### Subject selection

The protocol for this study was approved by the National Research Ethics Committee (London, UK) (Study Reference 13/LO/1509) and the Research and Development Office (NHS Ayrshire & Arran). Twenty patients with diabetes attending the Diabetes/Podiatry clinic at University Hospital Ayr (NHS Ayrshire & Arran) were selected for this study and written consent was obtained. Patients were excluded if they had received antibiotic treatment in the past 3 months. All study participants were Caucasian, diagnosed with Type 2 diabetes and the group comprised of 18 males and 2 females with an age range of 51 to 86 years. Ten subjects were selected with a new ulcer (within 3 days of ulcer formation) and ten subjects were selected with a recurring ulcer (any secondary ulcer regardless of location) [44].

### Swab sample collection

During a routine visit to the podiatry clinic patients had their wound dressing changed and ulcer examined. At this time, standard protocols were followed to sample the total surface of the foot ulcer with a Regular FLOQSwab™ (COPAN, Brescia, Italy), which was directly placed into a tube containing 2 ml of sterile PBS and immediately placed on ice, for DNA isolation. A second swab (Sterilin, Thermo Scientific, UK) was then used to sample the same area of the wound and sent to the clinical laboratory at University Hospital, Crosshouse (NHS Ayrshire & Arran) for standard aerobic and anaerobic culture. Swab sampling was used as the preferred method of sampling in this study as it is a less invasive alternative to ulcer biopsy and limits the risk of introducing infection in this vulnerable group of patients. Two control swabs were also was taken of the healthy skin on the unaffected foot of each patient. Hospital labs gave results in the form of presence or absence of species or groups of bacteria. One swab was used for DNA isolation and the other was assessed by aerobic and anaerobic culture to discount any bacterial strains, which may be part of the patient’s normal healthy skin flora. Information on current HbA1c levels and the duration of the diabetes were also taken from the patient. A Standard PCR was performed to determine the presence or absence of methicillin-sensitive *Staphylococcus aureus (*MSSA) and methicillin-resistant *S. aureus* (MRSA) using primers and conditions previously described by our group [45]. Primer sequences were as follows:

- *S. aureus*: F- ATTTGGTCCCAGTGGTGTGGGTAT,
- R-GCTGTGACAATTGCCGTTTGTCGT,
- *S. aureus mecA* primers: F- AACCACCCAATTTGTCTGCC,
- R- TGATGGTATGCAACAAGTCGTAAA.

### DNA isolation and purification

The DNA was isolated from bacterial cells carried on each FLOQSwab™ within 2 h of clinical sample collection. Bacterial cells were released from FLOQSwabs™ into the 2 ml of PBS by sonicating the samples in a water bath (Fisherbrand, ThermoFisher Scientific Inc., Loughborough, UK) at 35 kHz for 3 × 20s, and vortexing for 10s. The samples were centrifuged for 5 min at 5223 × *g* to pellet the cells. DNA was then isolated from each sample using bead-beating combined with a QIAamp DNA Mini Kit (Qiagen, UK), according to the manufacturer’s instructions. DNA was stored at −20 °C until required. The concentration and integrity of purified DNA was measured by NanoDrop analysis (Thermo Scientific, UK). It was our initial intention in this study to analyse the microbiome of the healthy control skin of each individual to compare with the microbiome of each ulcer. However, the concentration of DNA isolated from control samples was extremely low (in the range 0.5-1.27 ng/μl) and was much lower than the concentration required for sequencing (20 ng/μL), therefore these samples were removed from the study and control samples were analysed by traditional laboratory culture only.

### 16S amplicon sequencing (16S AS) and sequence analysis

16S AS was performed by GATC Biotech AG (Konstanz, Germany). DNA samples (total volume 30 μl [20 ng/μL]) were submitted for initial PCR amplification using primers for the V4 region of the 16S rDNA gene (515 F- AGAGTTTGATCCTGGCTCAG and 806R ATTACCGCGGCTGCTGG), producing a 253 bp product for sequencing. The Illumina HiSeq 2500 platform (Illumina Inc., San Diego, USA) was used to produce paired-end sequence reads using cyclic reversible chain termination chemistry. The base call of each sequence read was inspected and filtered for quality. Sequences below the quality threshold or less than 137 bp in length were removed. Sequence read pairs were merged using FLASh [46] and compressed based on 99 % similarity, using the clustering program CD-HIT-hit [47]. Chimeric clusters were removed using UCHIME [48], and unique clusters were subjected to BLASTn [49] analysis. Good quality and unique 16S rDNA sequences were used as a reference database to assign operational taxonomic unit (OTU) status to the clusters. Classification of OTU clusters and the number of reads within were consolidated to compute the relative abundance of each species within each sample.

#### Statistical analyses

Spearman’s rank correlation was used to assess correlation between culture and PCR results using GraphPad Prism software version 6.

OTU datasets were reduced by log2 transformation so as to carry out principal component analysis (PCA) and diversity statistics (Shannon diversity index and Dominance index); the analysis was carried out using PAST software [50].

An unpaired t-test was applied to compare diversity statistics and ulcer bacterial characteristics (oxygen tolerance, bacterial morphology, and number of OTU) using GraphPad Prism® software version 6. PCA was used to reduce the dimensionality of the OTU dataset. A scree plot was used to determine how many components emerged. No distinct clusters appeared between the two groups. Sample 13 was distant from the other samples and may have been skewing the data. Therefore analysis was repeated without Sample 13. To determine if distinct clusters formed for each group on the PCA plots, new variables were created for each principle component by using the factor loadings as regression coefficients, producing a score for each sample. These scores were then used as outcome variables to compare between groups.

The contribution of each bacterial class and genera that represented over 1 % of the group was calculated in terms of proportion to the overall sample, percentages were log transformed and an unpaired *t*-test was used to compare new and recurrent groups.

Characteristics of the ulcers (bacterial morphology, Gram type, oxygen tolerance, diversity levels, and common genera) were correlated to clinical aspects of the patient (HbA1c levels and duration of diabetes) using two-tailed Spearman’s correlation in GraphPad Prism.

## Availability of data and materials

Raw data sets are available from the following website: http://www.ncbi.nlm.nih.gov/ bioproject/304940.

## Abbreviations

16S AS: 16S amplicon sequencing
CoNS: coagulase negative staphylococci
OTU: operational taxonomic unit
